# Prevalence and correlates of substance use among undergraduates in a developing country

**DOI:** 10.4314/ahs.v21i2.49

**Published:** 2021-06

**Authors:** Chinyere Mirian Aguocha, Emeka Nwefoh

**Affiliations:** Department of Medicine, Imo state University, Owerri, Imo state

**Keywords:** Substance use, undergraduates, Nigeria

## Abstract

**Background:**

Psychoactive substance use is a major global public health issue. Use of psychoactive substances has been associated with negative consequences among students.

**Objective:**

The study assessed the prevalence and socio-demographic correlates of psychoactive substance use among undergraduate students in a Nigerian university.

**Materials and Methods:**

This was a cross-sectional descriptive study of 763 undergraduate students of Imo State University, Owerri, Nigeria, recruited using multi-stage sampling technique. Data on the socio-demographic characteristics and pattern of psychoactive substance use were collected using a structured questionnaire.

**Results:**

The lifetime rate of psychoactive substance use was 84.5%. Alcohol had the highest rate of lifetime (82.5%) and 12-month (61.1%) use. There was a similar rate of lifetime use of psychoactive substances among males (86.1%) and females (83.4%). Age (p<0.05) and place of residence (p<0.05) were significantly associated with lifetime psychoactive substance use. Catholics (OR:1.43; 1.03 – 1.99), whose friend (OR:1.94; 1.39 – 2.71), roommate (OR:3.06; (1.62 – 5.78) or brother (OR:1.22; 0.77 – 1.93) uses psychoactive substances were significantly more likely to have used substances in the past 12-months.

**Conclusion:**

There is a high rate of psychoactive substance use among the students. Age, religion, place of residence, family and peer use of substances are important determinants of psychoactive substance use.

## Introduction

Psychoactive substances are a class of substances, licit and illicit, that when ingested or administered affect mental processes.^1^ Psychoactive substance use is a major global public health issue, with an estimated 30 million substance users worldwide and most of these young people.^1^ Nigeria with its huge population of young people, ranks among one of the highest users of harmful drugs such as alcohol, tobacco, cannabis, benzodiazepines, cocaine, and opioids.^2^ Excessive use and uncontrolled access, coupled with concern over the link between psychoactive substance use with interpersonal relationships and cognitive difficulties, risky behavior, crimes and cultism among students have led to worries among both health personnel and educators.^3^–^6^

In Nigeria, the South East geopolitical zone (especially Imo State) is reported to have one of the highest prevalence rates of substance use in Nigeria.^7^

Among undergraduate students in Nigeria, the rate of psychoactive substance use is reported to be far higher than what is reported among the general populace.^7^,^8^,^9^,^10^ In Southwest Nigeria, 58.4% – 65% of undergraduates have used psychoactive substances at least once in their lifetime while 15.4% have reported current use.^8^,^9^ In Benin, South South Nigeria 46.6% lifetime rate has been found.^10^ Many studies show that alcohol is the most commonly used psychoactive substance among undergraduates. In Uyo, South South Nigeria, all the students surveyed reported a lifetime use of alcohol.^11^ In Southwest Nigeria, a wide range (13.6%–63.4%) was reported.^8^, ^9^, ^12^ In South East Nigeria, a range, (50.2%–78.4%) which showed a very high rate of current use of alcohol was found.^13^, ^14^ In North Central Nigeria, 77% lifetime rate was found in an early study^15^ but 15 years later, a lower rate of 13.6% was reported.^16^

Similarly, varying rates have been reported for other psychoactive substances. A study in South West Nigeria showed that tobacco (81%) was the commonest substance used.^17^ In North Central Nigeria^15^, ^16^, Southwest Nigeria^8^, ^9^, ^12^ and South East Nigeria, 3.2%–37.4%, 2.3%–43.8% and 12.1%^13^ respectively was reported. For sedatives, 6.1%–7.3% was found.^8^, ^16^

Relatively low use has been recorded for cannabis, organic solvents, hallucinogens, cocaine and narcotic analgesics.^15^ Marijuana use was found to be 1.4% in South East Nigeria^13^ while in South West Nigeria, 20% was reported.^12^ For stimulants, 45.3% and 29.1% lifetime and current use, 69.2% and 15.6% lifetime rates was reported in South East, North Central and Southwest Nigeria respectively.^8^, ^15^, ^18^

Findings on the association between gender and substance use have been mixed. In Enugu, South East Nigeria a marked disparity in the pattern of use was reported, which when disaggregated by gender showed male and female students respectively used alcohol 92.5% and 7.5%, tobacco (smoke) 96.1% and 1.0%) and marijuana 100.0% and 00.0%.^13^ A more recent study in the same region found that male undergraduates were five times more likely to use psychoactive substances compared to their female counterpart.^18^ In South West, Nigeria, it was found that lifetime use of alcohol was significantly higher among the male students (45.9%) compared to the female students (32.8%); current use of alcohol was significantly higher among the female students (16.4%) compared to the male students (10.2%).^9^ In South South Nigeria, a greater rate among females was reported.^11^

A study in Sudan found the overall prevalence of substance use to be 31%. The most common substance used was tobacco followed by cannabis and alcohol.^19^ The commonest reason for initiating substance use was curiosity.19 Peers were found to be the commonest sources of substances.^19^ The study found no association between age and substance use.^19^ Another study carried out among Ethiopian undergraduates found more substance use among children of uneducated parents.^20^ The same study found that older students were 2.4 times more likely to use substances than the younger students.^20^ Male students and students who have friends and family members who use substances were more likely to use substances.^20^

With the high rate of substance use reported in the state, it has become imperative to study the pattern and rates of substance use in one of the most highly populated high institutions located in the heart of the state. It is hoped that this paper will draw the attention of the relevant authorities to issue of substance use in the institution.

The aim of the study was to determine the prevalence and socio-demographic correlates of substance use among students of Imo State University, Owerri.

## Subjects and Methods

The study was carried out among undergraduate students of Imo State University, Owerri, Imo State. The University has 11 Faculties and 63 Departments. The university has a population of over fifteen thousand undergraduate students.

This was a descriptive cross-sectional study carried out on seven hundred and sixty three undergraduates who gave consent to be part of the study. Students were included in the study if they were current undergraduate students of IMSU who gave consent to be part of the study and were aged between 18–30 years. Students were excluded if they declined to take part and if they were absent from studies during data collection.

### Data collection

The sample size was calculated using 5% of the total estimated population of 15,000 current undergraduate students, and adjusting for 10% attrition, a total minimum sample size of 825 was achieved.^21^

Multi-stage sampling technique was used for sample selection. The 11 faculties of the Imo State University Owerri served as the sampling frame. During the first stage, for ease of data collection, using simple random sampling, 3 faculties were selected. These were the faculties of Business Administration, Education, and Social Sciences.

During the second stage, using simple random sampling, 2 departments were selected from each faculty. From the Faculty of Business Administration, the Departments of Management and Insurance were selected, from Faculty of Education, the Departments of Education Biology and Education History were selected while from the Faculty of Social Sciences, the Departments of Psychology and Mass Communication were selected.

In the third stage, using proportionate sampling techniques, respondents were recruited from each department to make up the calculated sample size. Each department was stratified by years of study and gender, on the assumption that duration of stay in the campus and sex would affect substance use. The total sample size was distributed proportionally to the selected departments based on the total number of students in each year of study according to gender. Finally, individual students fulfilling the inclusion criteria were randomly selected to be study participants.

Data collection took place between February and May 2019. A Structured questionnaire was used for data collection. The structured questionnaire was divided into two sections. Section A comprised the socio-demographic characteristics while Section B covered the relevant desired information for the study. Socio-demographic characteristics such as sex, age, marital status, religion, place of residence, parental employment status and level of education. Section B focused on substance use. This was partly adapted from the World Health Organization (WHO) model core student and focused on substances that are commonly used in Nigeria.22 Alcohol, Amphetamine, Cannabis, Cigarette, Codeine, Cocaine, Heroin, Sedatives, Solvents, and Tramadol were the substances studied. The non-existent drug “Relevin” was included as a validity check.^23^

The recruitment took place in the lecture halls. The sample frame consisted of students in the selected departments who were in the lecture hall, who volunteered to be part of the study and who met the inclusion criteria. Numbers were written on pieces of papers, which were then rumpled. The papers were shaken and were picked until the calculated sample size was achieved. Then the questionnaire was distributed to all the respondents. Guide on how to answer each question on the questionnaire was demonstrated to the students. To ensure confidentiality, the respondents were advised to fill the questionnaires when alone and a date was scheduled with them on when the questionnaires were to be returned to the researcher. Substance utilization was estimated for lifetime, 12 month and 30 day and 7 day use. For the purpose of this study, lifetime, 12 month and 30 day and 7 day use was defined as the use of substances at least once within the designated period.

Data handling: Data collected were cleaned manually and analyzed using computer software (SPSS for Windows, Version 21.0, Chicago, SPSS Inc.). Statistical testing was conducted at the 5% significance level. Socio-demographic characteristics were tabulated and frequencies and percentages calculated. Bivariate and multivariate analyses were conducted using Pearson's chi-square test and logistic regression to test the associations between variables.

### Ethical issues

Ethical clearance was obtained from the Ethics Committee of Imo State University Teaching Hospital with Letter of Introduction from Imo State University, Owerri. All respondents gave voluntary informed consent before recruitment into the study. This study did not involve any procedure that was detrimental to the respondent. It was explained to them that the information given would be kept confidential.

## Results

Out of the 825 questionnaires distributed, thirty seven questionnaires were not returned while 25 questionnaires were discarded due to inconsistent responses and recording the use of the non-existent drug Relexin.

Table 1 shows the socio-demographic distribution of the respondents.

**Table 1:** Socio-demographic characteristics of the respondents

Variable	Frequency N=763	Percentage (%)
**Gender**		
Male	323	42.3
Female	440	57.7
**Age (years)**		
18–22	494	64.7
23–26	226	29.6
27–30	43	5.6
**Marital status**		
Single	736	96.5
Married	27	3.5
**Religion**		
Catholic	305	40.0
Pentecostal	273	35.8
Anglican	156	20.4
Others	29	3.8
**Year in school**		
100	194	25.4
200	200	26.2
300	166	21.8
400	203	26.6
**Place of residence**		
Off-campus	478	62.6
Lives with parents	140	18.3
School hostel	98	12.8
Lives with relations	47	6.2
**Friends that use substances**		
Yes	433	56.7
No	330	43.3
**Father's employment status**		
Employed	608	79.7
Retired	99	13.0
Unemployed	40	5.2
Late	16	2.1

There were a total of 763 respondents, 440 females (57.7%) and 323 (42.3%) males. Most of the respondents (64.7%, N = 494) were aged 18–22 years, and were single, (96.5%, N=736). Many, (40%, N=305), belonged to the Catholic Church. Many of the respondents, (26.6%, N=203), were in fourth year. Most of the respondents, (62.6%, N=478) lived off-campus. Majority, (56.7%, N=433) had friends who used psychoactive substances.

Table 2 shows the rate of use of substances. The lifetime, 12-month, 30-day and 7-day use of substances was 84.5%, 63.0%, 45.9% and 28.2% respectively. The commonest substance used was alcohol with lifetime, 12-month, 30-day and 7-day rates of 82.5%, 61.1%, 44.2%, and 26.2% respectively. For lifetime use, the licit substances alcohol (82%), sedatives (14.2%), cigarette (13.6%), and tramadol (8.8%) were the commonest substances used while cannabis (7.6%) was the commonest illicit substance used.

**Table 2:** Rates of psychoactive substance use

Variable	Lifetime use	12 month	30 days	7 days
	n (%)	n (%)	n (%)	n (%)
**Substance** **use**	645(84.5)	481(63.0)	350 (45.9)	215 (28.2)
**Alcohol**	631(82.7)	466 (61.1)	337 (44.2)	200 (26.2)
**Sedatives**	108 (14.2)	59 (7.7)	40 (5.2)	20 (2.6)
**Cigarette**	104 (13.6)	54 (7.1)	42 (5.5)	35 (4.6)
**Tramadol**	67 (8.8)	56 (7.3)	30 (3.9)	18 (2.4)
**Cannabis**	58 (7.6)	37 (4.8)	37 (4.8)	27 (3.5)
**Codeine**	47 (6.2)	21 (2.8)	14 (1.8)	5 (0.7)
**Cocaine**	11(1.4)	4 (0.5)	-	-
**Solvents**	6 (0.8)	-	-	
**Amphetamine**	6 (0.8)	5 (0.7)	2 (0.3)	2 (0.3)
**Heroin**	4 (0.5)	3 (0.4)	-	-

N=763

Table 3 shows the determinants of lifetime substance use. Factors significantly associated with lifetime use of psychoactive substances were age (p<0.05), place of residence (p<0.05), use of psychoactive substances by friends, roommates, brother, sister, or a parent (p<0.05). Students who had friends who abused psychoactive substances were significantly about three time likely to have used substances (OR:3.27; 2.06 -5.18) compared to their counterparts.

**Table 3:** Determinants of lifetime substance use

Variable	Substance use		Stat	p-value	OR:95% CI
	Yes (%)	No (%)			
**Gender**					
Male	278 (86.1)	45 (13.9)			0.95 (0.61 – 1.48)
Female	367 (83.4)	73 (16.6)	χ2=1.01	0.18	1.00
**Age (years)**					
18–22	405 (82.0)	89 (18.0)			1.41 (0.55– 0.65)
23–26	205 (90.7)	21 (9.3)			2.33 (0.85 – 6.41)
27–30	35 (81.4)	8 (18.6)	χ2=9.37	0.01*	1.00
**Current marital status**					
Single	621 (84.4)	115 (15.6)			0.57 (0.15 – 2.21)
Married	24 (88.9)	3 (11.1)	χ2=0.41	0.52	1.00
**Religion**					
Catholic	264 (86.6)	41 (13.4)			1.30 (0.84 – 2.01)
Others	381 (83.2)	77 (16.8)	χ2=1.59	0.21	1.00
**Year in school**					
100	163 (84.0)	31 (16.0)			1.54 (0.87 – 2.75)
200	168 (84.0)	32 (16.0)			1.41 (0.81 -2.46)
300	148 (89.2)	18 (10.8)			1.88 (0.98 – 3.59)
400	166 (81.8)	37 (18.2)	χ2=3.98	0.26	1.00
**Place of residence**					
Lives with parents	110 (78.6)	30 (21.4)			1.00
Others	535 (85.9)	88 (14.1)	χ2=4.66	0.03*	1.41 (0.86 – 2.31)
**Father's employment** **status**					
Employed	508 (83.6)	100 (16.4)			1.08 (0.23 – 5.08)
Unemployed	33 (82.5)	7(17.5)	F=3.84	0.26	0.84 (0.14 – 4.89)
Retired	90 (90.9)	9 (9.1)			2.04 (0.38 – 11.10)
Late	14 (87.5)	2 (12.5)			1.00
**Use by friends**					
Yes	399 (92.1)	34 (7.9)			3.27*(2.06 -5.18)
No	246 (74.5)	84 (25.5)	χ2=44.4	<0.001*	1.00
**Use by room mate**					
Yes	113 (95.8)	5 (4.2)			2.56 (0.93 – 7.04)
No	532 (82.5)	113 (17.5)	χ2=13.5	<0.001*	1.00
**Use by brother**					
Yes	181 (91.4)	17 (8.6)			1.23 (0.63 -2.38)
No	464 (82.1)	101 (17.9)	χ2=9.7	<0.001*	1.00
**Use by sister**					
Yes	68 (93.2)	5 (6.8)			1.18 (0.37 – 3.74)
No	577 (83.6)	113 (16.4)	χ2=4.6	0.04*	1.00
**Parental use**					
Yes	138 (90.2)	15 (9.8)			0.98 (0.50 – 1.95)
No	507 (83.1)	103 (16.9)	χ2=4.7	0.02*	1.00
**Parental fights**					
Yes	37 (94.9)	2 (5.1)			2.27 (0.51 – 10.04)
No	608 (84.0)	116 (16.0)			1.00

* significant at p.0.05

F=Fisher's Exact Test

Table 4 shows the determinants of 12-month substance use. Gender, age, year in school, and father's employment status (p<0.05) were significantly associated with substance use. Use by friends, roommate, brother, sister, parent and frequent parental fights (p<0.05) were also significantly associated with 12-month psychoactive substance use.

**Table 4:** Determinants of 12 month substance use

Variable	Substance use		Stat	p-value	OR:95% CI
	Yes (%)	No (%)			
**Gender**					
Male	225 (69.7)	98 (30.3)			1.28 (0.91 – 1.79)
Female	256 (58.2)	184 (41.8)	χ2=10.5	<0.001*	1.00
**Age (years)**					
18–22	283 (57.3)	211(42.7)			0.84 (0.38 – 1.84)
23–26	168 (74.3)	58 (25.7)			1.22 (0.54 – 2.75)
27–30	30 (69.8)	13 (30.2)	χ2=20.2	<0.001*	1.00
**Current marital status**					
Single	463(62.9)	273 (37.1)			0.80 (0.32 – 2.00)
Married	18 (66.7)	9 (33.3)	χ2=0.2	0.69	1.00
**Religion**					
Catholic	205 (67.2)	100 (32.8)			1.43* (1.03 – 1.99)
Others	276 (60.3)	182 (39.7)	χ2=3.8	0.05	1.00
**Year in school**					
100	106 (54.6)	88 (45.4)			0.72 (0.46 – 1.14)
200	116 (58.0)	84 (42.0)			0.79 (0.51 – 1.21)
300	124 (74.7)	42 (25.3)			1.56 (0.96–1.55)
400	135 (66.5)	68 (33.5)	χ2=18.8	<0.001*	1.00
**Place of residence**					
Lives with parents	80 (57.1)	60 (42.9)			1.00
Others	401 (64.4)	222 (35.6)	χ2=2.6	0.11	1.09 (0.73 – 1.63)
**Father's employment** **status**					
Employed	369 (60.7)	239 (39.3)			0.64 (0.40 – 1.02)
Unemployed	100 (71.9)	39 (28.1)	χ2=6.1	0.01*	1.00
**Use by friends**					
Yes	315 (72.7)	118 (27.3)			1.94* (1.39 – 2.71)
No	166 (50.3)	164 (49.7)	χ2=40.9	0.001*	1.00
**Use by roommate**					
Yes	103 (87.3)	15 (12.7)			3.06* (1.62 – 5.78)
No	378 (58.6)	267 (41.4)	χ2=35.2	<0.001	1.00
**Use by brother**					
Yes	148 (74.7)	50 (25.3)			1.22 (0.77 – 1.93)
No	333 (58.9)	232 (41.1)	χ2=15.7	<0.001*	1.00
**Use by sister**					
Yes	55 (75.3)	18 (24.7)			0.72 (0.34 – 1.51)
No	426 (61.7)	264 (38.3)	χ2=5.2	0.02*	1.00
**Parental use**					
Yes	115 (75.2)	38 (24.8)			1.38 (0.84 – 2.27)
No	366 (60.0)	244 (40.0)	χ2=12.1	<0.001*	1.00
**Parental fights**					
Yes	33 (84.6)	6 (16.4)			2.43 (0.95 – 6.20)
No	448 (61.9)	276 (38.1)	χ2=8.2	<0.001*	1.00

* significant at p.0.05

Students who were Catholics (OR:1.43; 1.03 – 1.99), whose friends (OR:1.94; 1.39 – 2.71), roommates (OR:3.06; (1.62 – 5.78) or brother (OR:1.22; 0.77 - 1.93) used psychoactive substances were more likely to use psychoactive substance compared to their counterparts.

## Discussion

A high rate of substance use was found in this study. The United Nations Office on Drugs and Crime (UNODC) had reported in 2018 that Imo state had substance use problems exceeding national estimates.^22^ The high rate reported in this study may be a reflection of this. It also indicates the worsening psychoactive substance use situation among undergraduates.

The rates of alcohol use in this study were twice the rates reported in a cross-sectional survey of drug use among the general Nigerian population.^7^ It was also higher than what has been previously reported^13^–^16^ This indicates a worsening trend. The high prevalence of alcohol use may be related to its widespread acceptability and availability in society.

Cannabis was the most common illicit substance used.^7^,^15^, ^22^ However, the rate of use was 2.5 times lower than the estimated 10.8% and 10.9% reported among the general Nigerian and Imo state population respectively.^22^ Reasons for this may need to be further evaluated in future studies.

The rate of psychoactive substance use was higher among the male students. This is similar to what has been previously reported among undergraduates and in the general population in Nigeria.^10^, ^13^, ^18^, ^22^, ^23^, ^24^ This may be attributed to cultural factors which make psychoactive substance use more acceptable in males, and more opportunities for male psychoactive substance use.^25^ The high rate of alcohol use found in this study among females and the lack of significant difference in the lifetime use of alcohol between the genders show that this trend may be shifting. The high rate of female psychoactive substance use has been attributed to changing societal attitudes toward women, female incursion into previously male-dominated professions and females succumbing to peer pressure due to loss of parental control as a result of living away from the family.^11^, ^26^ This is buttressed by some studies in Nigeria which found a greater rate of substance use among females.^9^,^11^ This may however, be attributed to peculiarities in cultural norms and gender socialization patterns in the acceptability of psychoactive substance use by women.^27^

The greatest use of psychoactive substances was seen among those aged 23–26 years. There is similar reports of greater use among older youths.^10^, ^11^, ^17^, ^18^, ^28^ A general population study conducted in Nigeria found that the overall past-year use of most psychoactive substances was more among people within the age brackets of 25 and 39 years^22^ but in our study there was a reduced rate among those aged 27–30 years. Greater maturity can account for this. However, there are conflicting reports of greater use of psychoactive substances among the younger age group^11^ with alcohol, cigarette, and cannabis use being more common among younger persons but cocaine, and heroin use being more common among older persons.^17^ These, may be due to methodological differences, differences in substances studied, affordability and local preferences.

Similar to what was found in this study, substance use has been found to be least among those who live with relations (parents and extended family) and greatest among those who live in university hostels and off-campus.^18^ A similar finding was reported in a study carried out in Sudan which highlights the protective role of the family against negative peer influence.^19^ The lower rate among those who live with their relations may be due to improved supervision, authoritative and nurturing roles provided by the relations as opposed to too much freedom experienced while living alone.^29^ More attention has to be focused on the specific conditions in the hostels that could account for these difference and how to minimize them.

The family has a powerful influence on shaping the attitudes, values and behavior of children.^30^ There was greater use of substances among those whose family members (parents and siblings) used substances.^11^, ^30^, ^31^ This could be due to students imitating the behavior of their parents. Parents who use substances cannot effectively counsel their children against the use of substances.^11^, ^29^

Use by peers predicted the use of drugs.^3^, ^11^, ^32^ Peer influence has been reported to top the list for reasons for substance use.^11^ Need to belong and to be accepted by peers are part of the reasons for this. It has been reported that the influence of opposite-gender friends may be stronger than of same-gender friends especially in young adulthood, when mixed-gender relationships become more central.^33^ This needs to be further explored.

The rates reported in this study are slightly different from what has been observed in countries outside of Africa where a markedly higher use of nicotine, marijuana, solvents and cocaine has been reported.^34^, ^35^ Unlike what we reported, gender, financial background and academic variables were important factors in the use of psychoactive substances.^34^ But similar to what we reported, there was a very high rate of alcohol use.^34^ These differences could be attributed to local preferences and population sampled.

## Limitations

The study was a cross-sectional non-population representative study. Its results cannot be generalized to the entire population. Also causality cannot be determined. Substance use was determined through self-report, so 100% reliability cannot be guaranteed due to possible recall bias.

## Conclusion

There is a high rate of psychoactive substance use among the students. Age, religion, place of residence, family and peer use of substances are important determinants of psychoactive substance use.
